# The application potential of iMSCs and iMSC-EVs in diseases

**DOI:** 10.3389/fbioe.2024.1434465

**Published:** 2024-07-29

**Authors:** Xin Zhou, Jinyu Liu, Feifeng Wu, Jueyi Mao, Yang Wang, Junquan Zhu, Kimsor Hong, Haotian Xie, Binbin Li, Xinying Qiu, Xiangbin Xiao, Chuan Wen

**Affiliations:** ^1^ Department of Pediatrics, The Second Xiangya Hospital of Central South University, Changsha, China; ^2^ Department of Obstetrics, The Second Xiangya Hospital, Central South University, Changsha, China; ^3^ Department of Cardiovascular, People’s Hospital of Jianyang, Jianyang, China

**Keywords:** mesenchymal stem cells, induced pluripotent stem cells, extracellular vesicles, immunoregulation, cell therapy

## Abstract

The immune system, functioning as the body’s “defense army”, plays a role in surveillance, defense. Any disruptions in immune system can lead to the development of immune-related diseases. Extensive researches have demonstrated the crucial immunoregulatory role of mesenchymal stem cells (MSCs) in these diseases. Of particular interest is the ability to induce somatic cells under specific conditions, generating a new cell type with stem cell characteristics known as induced pluripotent stem cell (iPSC). The differentiation of iPSCs into MSCs, specifically induced pluripotent stem cell-derived mesenchymal stem cells (iMSCs), hold promise as a potential solution to the challenges of MSCs, potentially serving as an alternative to traditional drug therapies. Moreover, the products of iMSCs, termed induced pluripotent stem cell-derived mesenchymal stem cell-derived extracellular vesicles (iMSC-EVs), may exhibit functions similar to iMSCs. With the biological advantages of EVs, they have become the focus of “cell-free therapy”. Here, we provided a comprehensive summary of the biological impact of iMSCs on immune cells, explored the applications of iMSCs and iMSC-EVs in diseases, and briefly discussed the fundamental characteristics of EVs. Finally, we overviewed the current advantages and challenges associated with iMSCs and iMSC-EVs. It is our hope that this review related to iMSCs and iMSC-EVs will contribute to the development of new approaches for the treatment of diseases.

## 1 Introduction

The immune system is characterized by its ability to discern between “self” and “non-self”, fostering natural immune tolerance to the body’s own components while mounting immune responses to eliminate foreign substances. This capability is crucial for maintaining the physiological equilibrium and stability of the body. Imbalances in immune responses, whether excessive or deficient, can give rise to various diseases, including autoimmune disorders, immunodeficiency diseases, tumors (Parkin and Cohen, 2001). However, due to the intricate nature of the mechanisms underlying immune-related diseases and the limitations and adverse reactions associated with traditional therapies (Postal et al., 2016; Yu et al., 2023), there is an imperative to seek alternatives to conventional treatments.

Mesenchymal stem cells (MSCs), a versatile type of stem cells with the ability to support hematopoiesis (García-García et al., 2015) and differentiate into various cell types such as osteoblasts, chondrocytes, and adipocytes (Lin et al., 2019; Kim P. et al., 2022), were originally identified in the bone marrow and have since been isolated from a host of organs and tissues (Seok et al., 2020; Xie et al., 2022; Wei et al., 2023; Zhang et al., 2023). On top of all that, since both “mesenchymal stem cell” and “mesenchymal stromal cells” can be abbreviated as “MSC”, the current naming controversy primarily focuses on the terms “stem” and “stromal”. Hence, it is indispensable to state that the term “mesenchymal stem cell” should not amount to “mesenchymal stromal cell”. The International Society for Cell & Gene Therapy has proposed a minimal criteria to define “mesenchymal stromal cell” with markedly immunomodulatory, secretory, and homing properties as being plastic adherent, expressing CD73, CD90 and CD105, lacking the expression of hematopoietic and endothelial markers CD11b, CD14, CD19, CD34, CD45, CD79a and HLA-DR and capable of *in vitro* differentiation into adipocyte, chondrocyte and osteoblast lineages (Viswanathan et al., 2019). However, their “stemness” have not been clearly defined and may not meet the true standards of stem cells. The term “mesenchymal stem cell” was proposed by Caplan in 1991 to describe a group of progenitor cells with capabilities of self-renewal and differentiation (Viswanathan et al., 2019), which own unlimited proliferative potential and can be induced *in vitro* to differentiate into various mesodermal phenotypes and tissues. Bone marrow, periosteum, and connective tissue are their main tissue reservoirs (Caplan, 1991; Caplan, 2017). Regarded as a highly adaptable “all-around player”, MSCs exhibit low immunogenicity and possess immunomodulatory properties, making them promising candidates for addressing immune-related disorders, degenerative diseases, and traumatic injuries (Andrzejewska et al., 2021; Roth et al., 2022). In the area of stem-cell research, a significant milestone occurred in 2006 when Professor Shinya Yamanaka’s team at Kyoto University in Japan reported the groundbreaking induction of mouse fibroblasts into pluripotent stem cells, as published in the journal “Cell”. Utilizing a viral vector to introduce four transcription factors (*Oct3/4*, *Sox2*, *c-Myc*, and *Klf4*) into mouse fibroblasts, the team discovered that under conditions mimicking human embryonic stem cells (ESCs) culture, these cells could be prompted to transform into a new cell type exhibiting stem cell characteristics, termed induced pluripotent stem cells (iPSCs) (Takahashi and Yamanaka, 2006). Characterized by their high self-renewal capacity and pluripotent differentiation potential, iPSCs can differentiate into various cell types, including MSCs, neurons, and cardiomyocytes, under specific conditions. They find applications in drug development, medical aesthetics, and various other fields (Cyganek et al., 2018; Karagiannis et al., 2019; Okano and Morimoto, 2022; Bakhshandeh et al., 2023). Within this category, a type of MSCs derived from iPSCs through differentiation was defined in this paper as induced pluripotent stem cell-derived mesenchymal stem cells (iMSCs). As a novel stem cell type, their generation presents a unique opportunity to overcome existing barriers limiting the widespread application of MSCs in therapeutics, offering multiple potential alternative sources to traditional MSCs (Zhao et al., 2015; Spitzhorn et al., 2019; Rajasingh et al., 2021; Huang et al., 2022).

**FIGURE 1:**
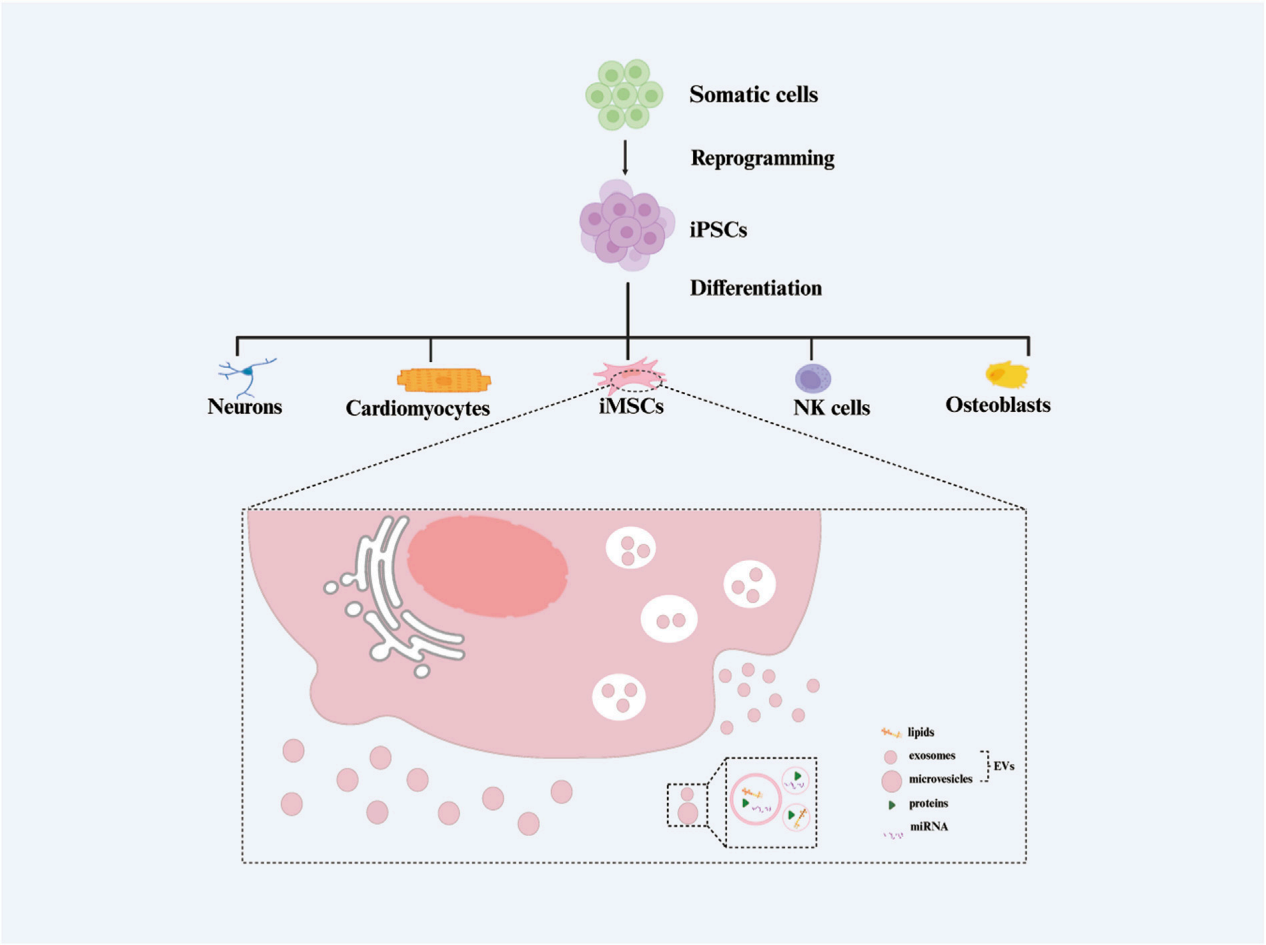
The generation process of extracellular vesicles isolated from induced pluripotent stem cell-derived mesenchymal stem cels (iMSC-EVs).

It is noteworthy that iMSCs bear the ability to secrete extracellular vesicles (EVs) (Ramos et al., 2022), referred to in this paper as induced pluripotent stem cell-derived mesenchymal stem cell-derived extracellular vesicles (iMSC-EVs) (Figure 1). These EVs can interact with various recipient cells, influencing diverse biological behaviors of target cells and thereby modulating physiological homeostasis and/or the progression of human diseases (Bertolino et al., 2022; Aldoghachi et al., 2023). EVs, nanosized vesicles secreted by various cells, have become a focal point in the field of cell therapy due to their advantages such as biocompatibility, safety, and ease of preservation. They hold the potential to transform conventional cell therapy into a “cell-free therapy” (Buzas, 2022).

In this review, we systematically reviewed the research progress of iMSCs and iMSC-EVs in diseases, summarized the biological effects of iMSCs on immune cells, highlighted the applications of iMSCs and iMSC-EVs in diseases, and briefly outlined the fundamental characteristics of EVs. Finally, we discussed the current advantages and challenges of iMSCs and iMSC-EVs in the treatment of immune-related disorders.

## 2 iMSCs

### 2.1 Roles of iMSCs in immunity

More and more evidence have indicated that iMSCs play a pivotal role in modulating immune responses. In the aspect of adaptive immunity orchestrated by T cells, numerous investigations have affirmed that iMSCs possess the capability to inhibit T cell proliferation, decrease Th1 and Th2 phenotypes, and upregulate Treg subsets. It has been further found that the administration of highly expressed soluble factors (TGF-β1/2/3, IL-10) in i-MSCs reduce the Th1, Th2 responses, upregulate the Treg response, and inhibit the cleavage of caspasse, suggesting that, the regulation of T cell responses by i-MSCs may be related to the above soluble factors secreted by i-MSCs to inhibit the cleavage of caspases (Li et al., 2017). There is a certain degree of controversy surrounding the immunomodulatory role of iMSCs in the Th17 cells. Some studies have reported that iMSCs can upregulate Th17 cells, while some researchers have shown that iMSCs can downregulate Th17 cells, and regulate the fate of CD4T lymphocytes from Th17 cells to Treg cell phenotype (Ng et al., 2016; Li et al., 2017; Wang et al., 2018). Additionally, iMSCs have been demonstrated a significant capacity to impede the proliferation, activation, and differentiation of cytotoxic CD8 T cells into Type 1 cytotoxic T cells (Tc1) and CD8 T cells expressing interleukin-17 (Tc17) (Wang et al., 2018). In the scope of innate immunity involving dendritic cells (DCs), iMSCs inhibit the differentiation of monocyte-derived DCs through the production of interleukin-10 (IL-10) and direct cells’ contacts. Furthermore, iMSCs do not impact the maturation of immature DCs into mature DCs; instead, they regulate their function via enhancing phagocytic capabilities and inhibiting the ability to stimulate lymphocyte proliferation (Gao et al., 2017). Concerning NK cells, iMSCs exert a significant suppressive effect on the proliferation and cytolytic function of NK cells, which are achieved by downregulating the expression of various activation markers and the ERK1/2 signaling pathway, resulting in impairing immune synapse formation and diminishing secretion of cytotoxic granules (Giuliani et al., 2011). As highlighted above, iMSCs primarily manifest immunosuppressive effects, suggesting a wide-ranging potential application in various diseases owing to their profound biological effects.

### 2.2 Diverse sources of iMSCs in various diseases

The current exploration of the efficacy and underlying mechanisms of iMSCs derived from various sources in conditions such as inflammatory diseases, cancer, and graft-versus-host reactions is steadily progressing (Fu et al., 2012; Hynes et al., 2013; Cheng et al., 2015; Liu B. et al., 2017; Wang et al., 2017; Fan et al., 2018; Hynes et al., 2018; Soontararak et al., 2018; Kagia et al., 2019; Yang et al., 2019; Zhong et al., 2019; Portier et al., 2021; Wang Z. et al., 2022). The section provided a comprehensive summary of recent research findings on iMSCs (Table 1).

**TABLE 1:** Summary of the recent progress of iMSCs in diseases.

No	Sources	Models	Assessment	Effects	References
1	N/A	Colitis mice model	Effects of MSCs from various sources in acute small intestine-colon inflammation	Prolong survival time by MSCs from various sources but failed to reduce inflammation through iMSCs	Kagia et al. (2019)
2	Fibroblasts	Colitis mice model	Effects of iMSCs and adMSCs in IBD	Ameliorate inflammation, increased the proliferation of IECs and Lgr5+ stem cells and, restore intestinal flora	Soontararak et al. (2018)
3	Fibroblasts	Colitis mice model	Effects and healing mechanisms of iMSCs in IBD	Promote healing by triggering the expression of TSG-6	Yang et al. (2019)
4	Renal tubular epithelial cells	Melanoma, breast cancer mouse models	Effects of *TRAIL-*iMSCs for targeted cancer therapy	Present strong anti-tumor effects on ranges of cancers	Wang et al. (2022a)
5	Fibroblasts	Melanoma mice model	Effects of *IL-*24- iMSCs with iMSCs in melanoma	Exhibit superior efficacy in restraining melanoma growth compared to iMSCs	Liu et al. (2017a)
6	PBMCs	Breast tumor mice model	Effects of *BRCA1*-deleted iMSCs on the progression of breast tumor	Promote angiogenesis, tumor growth and metastasis	Portier et al. (2021)
7	N/A	Chronic allergic airways inflammation mouse model	Effects of iMSCs and mechanisms in the immunomodulation	Prevent inflammation via TGF-β1 Smad2/Smad3 signaling pathway	Zhong et al. (2019)
8	N/A	Asthma mouse model	Involvement of lncRNA in iMSC treatment	Involve IncRNAs in iMSC therapy	Wang et al. (2017)
9	N/A	AR patients	Immunomodulatory effects of iMSCs	Modulate T-cell phenotype towards Th2 suppression through inducing Treg expansion	Fu et al. (2012)
10	N/A	AR patients	Immunomodulatory roles of iMSC on PBMCs	Activate quiescent T cells and elevated Treg cells response via NF-κB	Fan et al. (2018)
11	Fibroblasts	Periodontitis mice model	Effectiveness of iMSCs in Periodontitis	Control acute and chronic inflammatory responses associated with the destruction of periodontal tissue	Hynes et al. (2018)
12	Fibroblasts	Periodontal fenestration defect rat Model	Roles of iMSCs in the regenerative repair	Exhibit the capacity to regenerate periodontal tissues	Hynes et al. (2013)
13	Fibroblasts	Diabetic mice model	Efficacy of iMSC combined with Rapa in islet transplantation	Facilitate immune tolerance via suppressing Th1 and enhanced Treg cells differentiation	Cheng et al. (2015)

N/A, non-application; IBD, inflammatory bowel diseases; iMSCs, induced pluripotent stem cell-derived mesenchymal stem cells; IECs, intestinal epithelial cells; MSCs, mesenchymal stem cells; adMSCs, adipose-derived mesenchymal stem cells; TSG-6, tumor necrosis factor-α-stimulated gene 6; *TRAIL-*iMSCs, *TRAIL*-engineered induced pluripotent stem cell-derived mesenchymal stem cells; *IL*-24-iMSCs, *IL-24*-engineered induced pluripotent stem cell-derived mesenchymal stem cells; *BRCA1*, breast cancer susceptibility gene 1; AR, allergic rhinitis; lncRNAs, long non-coding RNAs; PBMCs, peripheral blood mononuclear cells; BM-MSCs, bone marrow derived-mesenchymal stem cells.

In an inflammatory bowel disease (IBD) model, Kagia et al. assessed the effectiveness of MSCs derived from bone marrow (BM-MSCs), umbilical cord blood (UCB-MSCs), human embryonic stem cells (ESC-MSCs), and iMSCs using a chemically induced acute small intestine-colon inflammation mouse model, and revealed that those types of stem cells significantly prolonged the survival time of experimental animals, although not all categories exhibited uniform improvements in the colon index. Both UCB-MSCs and BM-MSCs significantly reduced inflammation, while ESC-MSCs and iMSCs demonstrated either no improvement or only mild enhancement in intestinal inflammation post-treatment (Kagia et al., 2019). Likewise, Soontararak et al. compared the efficacy of iMSCs with adipose-derived MSCs (adMSCs) in a mouse model of IBD, finding that iMSCs were comparable to adMSCs in significantly ameliorating intestinal inflammation, notably increasing the population of intestinal Lgr5+ stem cells, and promoting intestinal vascularization. Encouragingly, following treatment with iMSCs or adMSCs, alterations in the microbial composition in colitis mice partially reverted to a profile resembling that of healthy mice (Soontararak et al., 2018). Yang et al. also delved into the regenerative potential of iMSC, shedding light on their capacity to foster mucosal healing by triggering the expression of tumor necrosis factor-α-stimulated gene 6 (TSG-6) (Yang et al., 2019).

Within the sphere of cancer research, especially when it comes to cancer treatment, *TRAIL*-engineered iMSCs (*TRAIL*-iMSCs) substantiated robust anti-tumor effects of *TRAIL*-iMSCs across various tumors both *in vivo* and *in vitro* (Wang Z. et al., 2022). And other scholars conducted a comparative analysis between the impacts of *IL-24* -engineered iMSCs (*IL-24-*iMSCs) and conventional iMSCs in melanoma, establishing that *IL24*-iMSCs exhibit superior efficacy in restraining melanoma growth compared to the control iMSCs (Liu B. et al., 2017). Besides, to explore how the absence or mutation of the breast cancer susceptibility gene 1 (*BRCA1*) influences tumor progression, researchers employed reprogramming techniques and formulated a model of breast cancer featuring *BRCA1* deletion, termed *BRCA1* ^+/−^iMSCs, revealing that, in contrast to *BRCA1* ^+/+^ iMSCs, *BRCA1* ^+/−^iMSCs prompted angiogenesis, culminating in tumor growth and metastasis (Portier et al., 2021).

Addressing airway inflammation, iMSCs displayed enduring capabilities to forestall chronic allergic airway inflammation through the potential mediation of the TGF-β1-Smad2/Smad3 pathway in orchestrating the immunoregulatory response of iMSC in chronic allergic airway inflammation (Zhong et al., 2019). Remarkably, a growing body of studies proposed a potential association about the modulation of long non-coding RNAs (lncRNAs) and T cell phenotypes involving in the mechanism underpinning iMSC therapy for allergic airway diseases (Fu et al., 2012; Wang et al., 2017; Fan et al., 2018).

In the domain of periodontal diseases, there is mounting evidence unveiling the efficacy of iMSCs in managing acute and chronic inflammatory responses linked to the deterioration and regenerative repair of periodontal tissues (Hynes et al., 2013; Hynes et al., 2018). As for transplantation, an investigation proposed that iMSCs possessed the attribution to facilitate immune tolerance by suppressing Th1 cells responses and promoting the differentiation of Treg cells (Cheng et al., 2015). In essence, these investigations collectively underscore the therapeutic promise of iMSCs across a spectrum of diseases.

Although MSCs have garnered significant attention and research in cell therapy, their plasticity results in different pathological microenvironments affecting their phenotype, immunogenicity, and immunomodulation in various ways (Liu et al., 2022; Tan et al., 2022). Consequently, it is essential to consider the impact of the body microenvironment on MSCs and how to enhance their clinical efficacy when applying MSCs for disease treatment. Regarding hypoxia, evidence indicates that even if MSCs initially have immune privilege, they eventually become immunogenic in the hypoxic environment of diseased tissues after infusion, leading to rejection by the host immune system. Both *in vivo* and *in vitro* experiments suggest that this may be due to hypoxia-induced upregulation of the 19S proteasome “Sug1”, downregulation of cyclooxygenase-2 (COX-2), and dysfunction of 26S proteasome assembly in MSCs (Abu-El-Rub et al., 2019; Abu-El-Rub et al., 2020; Sareen et al., 2020). Concerning inflammation, current research trends focus on the type and concentration of cytokines affecting MSCs. For example, the combined effect of TGF-β3 and BMP12 can induce a tenogenic differentiation of MSCs (Roth et al., 2018), while both TGF-β1 and TNF-α can synergistically induce the protein expression of CCL2, CXCL8, and COX-2, driving a pro-inflammatory fate in MSCs (Lerrer et al., 2017). When IFN-γ and TNF-α are present at 20 ng/mL or 50 ng/mL, MSC survival and differentiation are severely impaired, whereas at 5 ng/mL, MSCs not only maintain their viability and differentiation but also exhibit enhanced immunomodulatory properties (Barrachina et al., 2017a; Barrachina et al., 2017b). To improve the clinical efficacy of MSCs, researchers are addressing deficiencies by altering culture conditions before transplanting MSCs for treatment. For instance, preconditioning with hypoxia or inflammatory factors (such as IFN-γ, TNF-α, and IL-1β) can regulate innate and adaptive immune responses, including promoting M2 macrophage production, Treg cell proliferation, and reducing Th1 and Th17 cell proliferation (Saparov et al., 2016). With the advent of iMSCs, current research focuses on the therapeutic efficacy of the secretome from iMSCs preconditioned with hypoxia or inflammation (Wang S. et al., 2022), so the impact of hypoxia and inflammation on the phenotype, immunogenicity, and immunomodulation of iMSCs requires further exploration. Besides preconditioning *in vitro*, empowering cells through biomaterials and tissue engineering, nanotechnology, and genomic engineering, or finding substitutes for cell therapy, such as exosomes, conditioned medium, and apoptotic cell-derived vesicles, can further enhance the therapeutic effects of MSCs, holding the potential to achieve breakthroughs in MSC-based therapies (Ding et al., 2019; Adelipour et al., 2023; Chouaib et al., 2023; Zhu et al., 2023). As one of the substitutes for MSCs, iMSCs have garnered researchers’ attention for their iMSC-EVs in disease treatment and their conditioned medium in promoting skin wound healing (Liang et al., 2021). It is obvious that exploring the plasticity of MSCs and iMSCs and seeking innovative empowerment strategies and substitutes will be a crucial research direction (Najar et al., 2023).

## 3 iMSCs-EVs

### 3.1 Overviews of EVs

Extracellular vesicles are minute particles enveloped in a lipid bilayer, released by all types of cells. They harbor intricate cargos, encompassing nucleic acids like DNA, mRNA, and non-coding RNAs (ncRNAs), along with lipids and a variety of proteins, thereby facilitating intercellular communication (Mathivanan et al., 2010; Camussi et al., 2011; Azmi et al., 2013; Mashouri et al., 2019). On one hand, owing to their biological diversity, EVs are broadly categorized into two fundamental types: exosomes and ectosomes. On the other hand, considering their biophysical or biochemical traits, they can be further distinguished into small EVs (50–150 nm), medium EVs (200–800 nm), and large EVs (≥1000 nm) (Théry et al., 2018). As a cellular byproduct, EVs preserve the therapeutic effects of their parent cells, circumventing safety concerns associated with cell therapies (Kou et al., 2022). In addition, EVs can be purposefully engineered to deliver drugs or heighten drug sensitivity (Lou et al., 2015; Bagheri et al., 2020). In short, owing to their numerous advantages, such as biological compatibility and modifiability, EVs have emerged as an innovative strategy for disease treatment.

### 3.2 iMSC-EVs in diseases

EVs derived from iMSCs have exhibited considerable therapeutic potential across plentiful diseases (Hu et al., 2015; Zhang et al., 2015; Nong et al., 2016; Qi et al., 2016; Liu X. et al., 2017; Yuan et al., 2017; Zhu et al., 2017; Kim et al., 2018; Xia et al., 2020; Kim et al., 2021; Peng et al., 2021; Sun et al., 2021; Kim J. et al., 2022; Gao et al., 2022; Lang et al., 2023; Ye et al., 2023; Zhao et al., 2023; Kim et al., 2024) (Table 2). These discoveries underscore the promising therapeutic prospects of iMSC-EVs.

**TABLE 2:** Biological effects and mechanism of iMSC-EVs in diease models.

No	Models	Molecular mechanisms	Transferring materials	Target cells	Biological effects	References
**1**	SS mouse model	Decrease Th17 cells in the spleen, promoted M2 macrophage polarization by young iMSC-EVs	miR-125b inhibitors	Macrophages, Th17 cells	Impede onset of SS via youthful iMSC-EVs, restore effects on repressing sialadenitis onset by loading miR-125b inhibitors into aging iMSC-EVs	Zhao et al. (2023)
**2**	OA mouse model	N/A	N/A	Chondrocytes	Stimulate chondrocyte migration, proliferation and demonstrate a greater effect via iMSC-EVS than SMMSC-EVs	Zhu et al. (2017)
**3**	EAP rat model	Downregulate COX-2 overexpression, reinstated the Th1/Th2 and Treg/Th17 cells imbalances	N/A	Th1, Th2, Treg, Th17 cells	Ameliorate chronic pelvic pain and facilitate prostate tissue repair	Peng et al. (2021)
**4**	Tendinopathy rat model	Downregulate HIF-1 pathway	N/A	Mast cells	Alleviate tendon injury pain	Gao et al. (2022)
**5**	Tendinopathy rat model	Downregulate p38 MAPK pathway, regulate heterogeneity of macrophages	DUSP2, DUSP3	Macrophages	Mitigate pain in tendinopathy	Ye et al. (2023)
**6**	Renal I/R rat model	Activate SP1–SK1–S1P pathway	SP1	Kidney cells	Protect against R/I injury	Yuan et al. (2017)
**7**	Liver I/R rat model	Anti-apoptotic, antioxidative stress response role	N/A	Hepatocytes, macrophages, neutrophils	Alleviate liver I/R injury	Nong et al. (2016)
**8**	Skin wound rat model	N/A	N/A	Fibroblasts, endothelial cells	Promote collagen synthesis and angiogenesis	Zhang et al. (2015)
**9**	Skin wound model *in vitro*	Stimulate ERK1/2 pathway	N/A	HaCaT, HDFs	Promote proliferation of skin cells	Kim et al. (2018)
**10**	Hind-limb ischemia mouse model	Activate angiogenesis-related gene expression	N/A	Endothelial cells	Protect limbs from ischemic injury	Hu et al. (2015)
**11**	ONFH	Activate PI3K/Akt pathway	N/A	Endothelial cells	Promote vascular generation and prevent bone loss	Liu et al. (2017b)
**12**	Osteoporotic rat model	N/A	N/A	Osteoblasts	Promote vascular generation and regeneration of bone tissue	Qi et al. (2016)
**13**	Ischemic stroke rat model	Suppress autophagy via activation of STAT3 pathway	N/A	Endothelial cells	Promote angiogenesis and protect against ischemic brain injury	Xia et al. (2020)
**14**	DM-POCD mouse model	Inhibit EphA4, CDKN2C, FoxO1 expression	miR-21-5p, miR-486-5p	H-NSCs	Promote neurogenesis and restore cognitive function	Lang et al. (2023)
**15**	IVDD	Downregulate the cAMP specific hydrolase and activate Sirt6 pathway	miR-105-5p	NPCs	Rejuvenate senescent nucleus pulposus cells and attenuate intervertebral disc degeneration	Sun et al. (2021)
**16**	AD mice model	Inhibit Th2 cytokine receptors, NF-κB, IL-31R-STAT pathway, increase expression of skin barrier integrity-related genes and proteins	N/A	Th2, mast cells	Suppress inflammation and facilitate skin barrier restoration	Kim et al. (2022b)
**17**	AKI mice model	Relieve tissue inflammation and immune cell infiltration, anti-apoptosis	N/A	Kidney cells	Enhance kidney-protective function	Kim et al. (2024)
**18**	NASH mice model	Reduce inflammatory factors, ER and mitochondrial stress	N/A	Hepatocytes	Ameliorate the progression of NASH	Kim et al. (2021)

N/A, non-application; iMSCs, induced pluripotent stem cell-derived mesenchymal stem cells; iMSC-EVs, induced pluripotent stem cell-derived mesenchymal stem cell-derived extracellular vesicles; SMMSC, synovial membrane mesenchymal stem cells; EVs, extracellular vesicles; SS, Sjögren’s syndrome; OA, osteoarthritis; SMMSC-EVs, synovial membrane mesenchymal stem cell-derived extracellular vesicles; EAP, experimental autoimmune prostatitis; SP1, specificity protein 1; I/R, ischemia-reperfusion; HaCaT, human keratinocytes; HDFs, human dermal fibroblasts; ONFH, osteonecrosis of the femoral head; ERK, extracellular signal-regulated kinase; DM-POCD, diabetes mellitus-postoperative cognitive dysfunction; NPCs, nucleus pulposus cells; IVDD, intervertebral disc degeneration; AD, atopic dermatitis; AKI, acute kidney injury; NAFLD, non-alcoholic fatty liver disease; ER, endoplasmic reticulum.

In terms of autoimmune diseases and inflammatory responses, researchers posited that youthful iMSC-EVs, as opposed to aging counterparts, impeded the onset of Sjögren’s syndrome (SS) by bolstering M2 macrophages and diminishing Th17 cells. Also, countering miR-125b in aging iMSC-EVs could reinstate this function (Zhao et al., 2023). In an osteoarthritis (OA) model, administering iMSC-EVs and synovial membrane mesenchymal stem cell-secreted extracellular vesicles (SMMSC-EVs) both mitigated OA, yet the therapeutic efficacy of iMSC-EVs surpassed that of SMMSC-EVs. Similarly, both iMSC-EVs and SMMSC-EVs stimulated chondrocyte migration and proliferation, albeit iMSC-EVs demonstrated a more robust effect (Zhu et al., 2017). What’s more, iMSC-EVs harbored the potential to ameliorate chronic pelvic pain, alleviated voiding dysfunction, suppressed inflammatory responses, and facilitated prostate tissue repair. These functionalities could be induced through the downregulation of Cyclooxygenase-2 (COX-2) overexpression, reinstating the Th1/Th2 and Treg/Th17 cells imbalances (Peng et al., 2021). Additionally, iMSC-EVs present promise in alleviating tendon injury pain by impeding mast cell activation (Gao et al., 2022). Another study also indicated that iMSC-EVs could significantly mitigated pain in tendinopathy, but by regulating the heterogeneity of infiltrated macrophages, which was mediated *via* the p38 MAPK pathway through transporting DUSP2 and DUSP3. Thus, it might be a prospective candidate for tendinopathy therapy (Ye et al., 2023).

Increasingly, extensive researches unfolded that iMSC-EVs owned advantages in tissue injury, degeneration and cognitive dysfunction. In the context of tissue ischemia-reperfusion (I/R) injury, iMSC-EVs guarded against I/R injury by delivering specificity protein 1 (SP1) and transcriptional activation of sphingosine kinase 1, while inhibiting renal necrosis (Yuan et al., 2017). Others suggested that human iMSC-EVs could alleviate liver I/R injury by inhibiting inflammatory reactions, attenuating oxidative stress responses, and inhibiting cell apoptosis (Nong et al., 2016). In tissue reconstruction, iMSC-EVs promoted skin wound healing by enhancing collagen synthesis and vascularization (Zhang et al., 2015). To further explore their mechanisms, investigators emphasized that iMSC-EVs boosted extracellular signal-regulated kinase (ERK)-1/2 signaling pathway to promote the proliferation of skin cells (Kim et al., 2018). Apart from those, iMSC-EVs could not only promote vascular generation to alleviate limb ischemia and prevent bone loss toward deterring femoral head necrosis through the activation of the PI3K/Akt signaling pathway, but also play an osteogenic role in participating in bone tissue regeneration in osteoporotic rat model (Hu et al., 2015; Qi et al., 2016; Liu X. et al., 2017). Still, as regards nervous system, iMSC-EVs fostered angiogenesis after ischemic stroke by inhibiting autophagy, and STAT3 signaling pathway might took part in the process (Xia et al., 2020). Another study uncovered that iMSC-EVs transferred miR-21-5p and miR-486-5p to promote hippocampal neural stem cells (H-NSCs) proliferation and neurogenesis through inhibiting EphA4, CDKN2C, and FoxO1 expression in diabetes mellitus-postoperative cognitive dysfunction (DM-POCD) (Lang et al., 2023). As carriers, iMSC-EVs could deliver exogenous miR-105-5p, revitalizing aging nucleus pulposus cells (NPCs) and alleviating intervertebral disc degeneration (IVDD) via downregulating the cAMP specific hydrolase and activating the Sirt6 pathway (Sun et al., 2021). Notably, the biological roles of iMSC-EVs can be enhanced by preconditioning. One example was that EVs derived from IFN-γ-stimulated iMSCs (IFN-γ-iMSC-EVs) inhibited Th2-induced immune responses, suppressed inflammation and pruritus concerning the reduced activity of the NF-κB and IL-31R-STAT signaling pathway, and facilitated skin barrier restoration which might be involved in the increasement of expression of skin barrier integrity-related genes and production of lipid synthesis-related proteins in atopic dermatitis (AD) (Kim J. et al., 2022). Similarly, compared with iMSC-EVs applied directly to acute kidney injury (AKI), IFN-γ-iMSC-EVs displayed a more significantly protective effect, such as relieving tissue inflammation and immune cell infiltration, anti-apoptosis (Kim et al., 2024). For another example, researchers disclosed that EVs isolated from pan peroxisome proliferator-activated receptors agonist-stimulated iMSCs (pan PPAR-iMSC-EVs) ameliorated the progression of non-alcoholic fatty liver disease (NAFLD), reduced endoplasmic reticulum (ER) and mitochondrial stress, and promoted regeneration of hepatocytes (Kim et al., 2021). Altogether, iMSC-EVs represent a promising therapeutic approach for a variety of different diseases.

## 4 Advantages and challenges of iMSCs and iMSC-EVs

As previously mentioned, while iMSCs and iMSC-EVs hold promising potential in clinical applications, there remain challenges in transitioning them from the laboratory to the clinic. Following this, we delved into the current strengths and hurdles associated with the use of iMSCs and iMSC-EVs in the treatment of immune-related diseases (Table 3).

**TABLE 3:** Advantages and challenges of iMSCs and iMSC-EVs.

Challenges	Advantages
iMSCs	
• Early stage of industrialization • Controversial safety and ethics • Obscure therapeutic mechanisms and heterogeneity • Not true substitutes for BM-MSCs • High costs and technical requirements	• Unlimitied and personalized sources • Lower immunogenicity, tumorigenicity and avoiding ethical restrictions • Being more homogeneous than traditional MSCs • Scalable production, stable quality and better physiological effects
iMSC-EVs	
• Lack of standardization for efficient extraction, purification and storage strategies • Heterogeneity and low yield • Ambiguous mechanisms in treating diseases • Obscure biosafety, stability of cargos, optimal injection dose and distribution law in the body • Low drug-delivery efficiency	• A form of “Cell-free” therapy • Better physiological effects than traditional MSC-EVs • Emerging artificial EVs with higher delivery efficiency

MSCs, mesenchymal stem cells; iMSCs, induced pluripotent stem cell-derived mesenchymal stem cells; iMSC-EVs, induced pluripotent stem cell-derived mesenchymal stem cell-derived extracellular vesicles; MSC-EVs, mesenchymal stem cell-derived extracellular vesicles; BM-MSCs, bone marrow derived-mesenchymal stem cells.

### 4.1 Advantages and challenges of iMSCs

MSCs have lots of sources, primarily categorized as adult MSCs derived from bone marrow and adipose tissue, as well as embryonic tissue-related MSCs, such as umbilical cord MSCs (UC-MSCs) and placental MSCs (Seok et al., 2020; Xie et al., 2022; Wei et al., 2023; Zhang et al., 2023). However, MSCs from different origins exhibit certain heterogeneity (Yang et al., 2017; Wang et al., 2020). While this heterogeneity forms the basis for the diversity in their biological effects, it also introduces challenges for standardization and normalization in evaluating MSC efficacy. With the maturation of iPSCs reprogramming technology (Liuyang et al., 2023), our understanding of the molecular mechanisms maintaining stem cell “stemness” has deepened, opening up limitless possibilities for potential MSC sources. In theory, iPSCs possess the potential for multilineage differentiation and self-renewal, making iMSCs suitable for large-scale cultivation and overcome tissue-and age-related heterogeneity (Wruck et al., 2021). Moreover, in comparison to traditional tissue-derived MSCs, iMSCs exhibit greater stability and enhanced therapeutic efficacy in disease models. For instance, researchers induced iPSCs derived from human urothelial cells into mesenchymal stem cells, comparing their therapeutic effects in regenerative diseases with UC-MSCs. It was indicated that CD73, CD90 and CD105 gene transcripts and proteins were highly expressed in iMSCs and UC-MSCs, which were absent in other cells. Final results showed that even in later passages (P15), iMSCs maintained their MSC characteristics without any chromosomal abnormalities, whereas UC-MSCs began to lose their MSC characteristics during this period. Importantly, iMSCs demonstrated superior wound healing capabilities in migration assays compared to UC-MSCs (Marson et al., 2008). Unlike ESCs, iPSCs can be derived from the patient’s own cells (Portier et al., 2021), avoiding issues of immune rejection and ethical concerns. Furthermore, iMSCs can now be induced without viral vectors or the oncogenic gene *c-myc* (Marson et al., 2008; Yu et al., 2009), reducing the risk of tumorigenesis. Based on these characteristics, iPSCs can provide relatively stable, homogeneous and personalized iMSCs for specific patients, concurrently propelling the industrialization of MSCs. It’s noteworthy that iPSCs, as an emerging technology, are still in the early stages of industrial application. With regard to ethics and safety, while iPSCs can be derived from human somatic cells, thus avoiding the ethical issues associated with ESCs and reducing immune rejection, they also raise several ethical concerns, including informed consent, abnormal reprogramming, tumorigenicity, and the production of human germ cells (Zheng, 2016; Moradi et al., 2019). Obtaining informed consent is a major ethical issue in hiPSC research, requiring full consent from both cell donors and recipients. Aslo, many reprogramming mechanisms remain unknown, and abnormal reprogramming and stem cell proliferation can lead to tumor formation (Zheng, 2016). Generating human germ cells from iPSCs, despite the potential benefits, poses an ethical challenge because these germ cells could be used illegally and unethically in reproductive activities (Moradi et al., 2019). Therefore, the application of iPSCs in germ cells requires debate from legal, ethical, and practical perspectives. Furthermore, issues related to the immunogenicity, genetic instability, and dosage toxicity of iPSCs need further exploration and validation (Neofytou et al., 2015). For a more comprehensive comparison of the therapeutic effects between iMSCs and conventional tissue-derived MSCs, further research on their efficacy and mechanisms of action is necessary (Zhu et al., 2017). And even though iMSCs are an independent entity of MSCs with higher therapeutic potential than their donor-matched parent MSCS, it must be pointed out that there is significant functional heterogeneity among iMSCs, which is affected by the nature of the parental cells or generation protocols of iMSCs, demonstrating the importance of screening donors on an individual basis and standardizing iMSCs (Lee et al., 2023). It’s worth noting that, compared to conventional tissue-derived mesenchymal stem cells, some researchers suggested that, based on currently widely used preparation protocols, iMSCs were not true substitutes for BM-MSCs. Their studies indicated that, although iMSCs met the minimum standards for MSCs marker expression, they exhibited substantial differences in differentiation potential, with a significant reduction in chondrogenesis and adipogenesis. To expose the cellular basis behind these differences, researchers carried some comparisons between iMSCs and BM-MSCs. They found that, compared to BM-MSCs, iMSCs expressed very high levels of KDR and MSX2. Additionally, the PDGFRα level in BM-MSCs significantly increased. These distinct gene expression profiles remained constant during the culture expansion process, suggesting a closer relationship between the iMSCs and vascular progenitor cells (VPCs). The study highlighted that the high-level expression of typical MSC markers were insufficient to distinguish MSCs from other mesodermal progenitor cells (Xu et al., 2019). Finally, unlike the differentiation of ordinary cells, the effective differentiation of iPSCs into target cells requires higher technological barriers, demanding increased requirements for production processes and quality control (Zhang et al., 2022). Due to the additional differentiation steps required for iMSC generation, some researchers directly used undifferentiated iPSCs as sources of therapeutic EVs production in diabetic mouse models. Interestingly, their vascularization bioactivity was similar to donor-matched iMSC-EVs, while their anti-inflammatory bioactivity was superior to the latter (Levy et al., 2023). Despite the challenges associated with iMSCs, ongoing in-depth research in the iPSC field, gradual improvements in iMSC production techniques, and the industrial transformation of iMSCs are expected to accelerate, addressing the clinical needs of numerous refractory diseases.

### 4.2 Advantages and challenges of iMSC-EVs

As a form of “cell-free therapy”, EVs have emerged as potent weapons in treating immune-related diseases. Recently, researchers have reported the positive roles of iMSC-EVs and MSC-EVs in varying conditions, exploring their differences in therapeutic effects. For example, researchers have assessed the ability of both to promote skin cell proliferation, discovering that although both increased the vitality and cell cycle progression of human keratinocytes (HaCaT) and human dermal fibroblasts (HDFs), iMSC-EVs notably boosted the proliferation of HaCaT. Moreover, both EVs enhanced the secretion of collagen in HaCaT and HDFs, with an observed increase in fibronectin levels only in HaCaT, and this role was superior through the treatment of iMSC-EVs (Kim et al., 2018). In another study, researchers compared the efficacy of SMMSC-EVs and iMSC-EVs in treating osteoarthritis and detected that both of them could alleviate OA in a mouse OA model, but the therapeutic effect of iMSC-EVs surpassed that of SMMSC-EVs. Similarly, they promoted chondrocyte migration and proliferation, with iMSC-EVs exhibiting stronger effects (Zhu et al., 2017). In summary, these experiments indicate similarities in efficacy between the two EVs, but in specific effects, iMSC-EVs possess certain superiority, demonstrating their potential in cell-free therapy. However, iMSC-EVs and conventional mesenchymal stem cell-derived extracellular vesicles (MSC-EVs) share common issues, they still encounter bottlenecks in standardization, scaling, and heterogeneity that urgently need addressing. Firstly, systematic standards and protocols for the extraction, purification, and storage of MSC-EVs have not been established. Different cell sources and culture conditions also result in MSC-EVs subpopulations with distinct characteristics and biological functions (Luo et al., 2021; Almeria et al., 2022; Li et al., 2023; Tertel et al., 2023). Therefore, new standardized strategies for extracting, purifying, and storing EVs are needed to meet good manufacturing practice standards. Secondly, the efficiency of large-scale extraction of MSC-EVs is also a hurdle in the clinical translation of EVs. Despite the development of various effective isolation and purification technologies, the combined use of multiple technologies complicates the preparation process, and large-scale production still wait for new isolation and purification techniques (Dao et al., 2022). Thirdly, it is necessary to quantitatively and qualitatively determine the molecular spectrum of EVs to explore their specific mechanisms in diseases and evaluate their clinical potential as alternatives to cell therapy (Zhu et al., 2017). Fifthly, enhancing the efficiency of EV drug delivery is crucial. There is increasing focus on developing artificial EVs, which exhibit higher loading capacities and delivery efficiencies compared to traditional EVs (Lu and Huang, 2020; Li et al., 2021). More importantly, large-scale studies are still needed to further research the biosafety of MSC-EVs, the stability of their contents in the human body environment, optimal injection doses and their distribution patterns *in vivo*. Thus, transitioning iMSC-EVs to drugs involves unknown challenges, making this a potentially lengthy process. However, organizations like the International Society for Extracellular Vesicles (ISEV) have developed guidelines relatively detailing standard operating procedures that should be followed in the collection and quality control processes for EVs (Théry et al., 2018). With rigorous scientific research and effective regulation, significant breakthroughs are expected in this field.

## 5 Conclusion

iPSCs, a technology involving the introduction of a series of inducing factors into somatic cells, reprogramming them into cells with characteristics akin to ESCs, offer a fresh perspective for the clinical application and research of stem cells (Takahashi and Yamanaka, 2006). iMSCs, a product of iPSCs, not only offer widespread sourcing, abundance, good efficacy, and higher homogeneity but also address concerns related to immune rejection and ethical issues. Importantly, iMSC-EVs, as a derivative of iPSCs, might exhibit similar or even stronger efficacy compared to MSC-EVs. However, as described above, iMSCs, as a nascent technology, face several challenges regarding heterogeneity, complicate mechanisms of action, and demanding production processes. Simultaneously, EVs encounter bottlenecks in standardization, scaling, and heterogeneity and so on. Therefore, while iMSC and iMSC-EVs technology have made significant breakthroughs in recent years, their clinical application remains in early stages and warrants further exploration for the time to come.

## Figures and Tables

**Table:** 

**AD**	Atopic dermatitis
**AdMSCs**	Adipose-derived mesenchymal stem cells
**AKI**	Acute kidney injury
**BM-MSCs**	Bone marrow derived-mesenchymal stem cells
**BRCA1**	Breast cancer susceptibility gene 1
**COX-2**	Cyclooxygenase-2
**DCs**	Dendritic cells
**DM-POCD**	Diabetes mellitus-postoperative cognitive dysfunction
**ER**	Endoplasmic reticulum
**ERK**	Extracellular signal-regulated kinase
**ESC-MSCs**	Embryonic stem cell-derived mesenchymal stem cells
**ESCs**	Embryonic stem cells
**EVs**	Extracellular vesicles
**hiPSCs**	Human induced pluripotent stem cells
**H-NSCs**	Hippocampal neural stem cells
**I/R**	Ischemia-reperfusion
**IBD**	Inflammatory bowel diseases
**IECs**	Intestinal epithelial cells
**IFN-γ-iMSC-EVs**	EVs derived from IFN-γ-stimulated iMSCs
**IL-10**	Interleukin-10
***IL-24-*iMSCs**	*IL-24*-engineered-induced pluripotent stem cell-derived mesenchymal stem cells
**iMSC-EVs**	Induced pluripotent stem cell-derived mesenchymal stem cell-derived extracellular vesicles
**iMSCs**	Induced pluripotent stem cell-derived mesenchymal stem cells
**iPSCs**	Induced pluripotent stem cells
**ISEV**	International society for extracellular vesicles
**IVDD**	Intervertebral disc degeneration
**lncRNAs**	Long non-coding RNAs
**MSCs**	Mesenchymal stem cells
**N/A**	Non-application
**NAFLD**	Non-alcoholic fatty liver disease
**ncRNAs**	Non-coding RNAs
**NPCs**	Nucleus pulposus cells
**OA**	Osteoarthritis
**pan PPAR-iMSC-EVs**	EVs isolated from pan peroxisome proliferator-activated receptors agonist-stimulated iMSCs
**PBMCs**	Peripheral blood mononuclear cells
**qRT-PCR**	quantitative real time polymerase chain reaction
**SMMSC-EVs**	Synovial membrane mesenchymal stem cells-derived extracellular vesicles
**SP1**	Specificity protein 1
**SS**	Sjögren’s Syndrome
**Tc1**	Type 1 cytotoxic T cells
**Tc17**	IL-17-expressing-CD8 T cells
***TRAIL-*iMSCs**	*TRAIL*-engineered induced pluripotent stem cell-derived mesenchymal stem cells
**Treg cells**	Regulatory T cells
**TSG-6**	Tumor necrosis factor-α-stimulated gene 6
**UCB-MSCs**	Umbilical cord blood-derived mesenchymal stem cells
**UC-MSCs**	Umbilical cord-derived mesenchymal stem cells
**VPCs**	Vascular progenitor cells
